# Ginsenoside Rp1, A Ginsenoside Derivative, Augments Anti-Cancer Effects of Actinomycin D via Downregulation of an AKT-SIRT1 Pathway

**DOI:** 10.3390/cancers12030605

**Published:** 2020-03-05

**Authors:** Un-Jung Yun, In Hye Lee, Jae-Seon Lee, Jaegal Shim, Yong-Nyun Kim

**Affiliations:** 1 Division of Translational Science, National Cancer Center, 323 Ilsan-ro, Ilsandong-gu, Goyang-si 10408, Korea; yun2456@ncc.re.kr (U.-J.Y.); jaegal@ncc.re.kr (J.S.); 2 Department of Life Science, Ewha Womans University, Seoul 03760, Korea; lih3026@ewha.ac.kr; 3 Department of Molecular Medicine, College of Medicine, Inha University, Incheon 22212, Korea; 214007@inha.ac.kr

**Keywords:** actinomycin D, AKT, combination therapy, colon cancer, ginsenoside Rp1, multidrug resistance, SIRT1

## Abstract

Novel strategies for overcoming multidrug resistance are urgently needed to improve chemotherapy success and reduce side effects. Ginsenosides, the main active components of *Panax ginseng*, display anti-cancer properties and reverse drug resistance; however, the biological pathways mediating this phenomenon remain incompletely understood. This study aimed to evaluate the anti-cancer effects of ginsenoside Rp1, actinomycin D (ActD), and their co-administration in drug-resistant cells and murine xenograft model of colon cancer, and explore the underlying mechanisms. ActD increased expression and activity of SIRT1 in drug-resistant LS513 colon cancer, OVCAR8-DXR ovarian cancer, and A549-DXR lung cancer cells, but not in ActD-sensitive SW620 colon cancer cells. Inhibition of SIRT1, either pharmacologically, with EX527 or through siRNA, stimulated p53 acetylation and apoptosis in LS513 cells when treated with ActD. ActD also increased AKT activation in drug-resistant cells. Inhibition of AKT abrogated ActD-induced upregulation of SIRT1, suggesting that the AKT-SIRT1 pathway is important in ActD resistance. Rp1 inhibited both ActD-induced AKT activation and SIRT1 upregulation and re-sensitized the cells to ActD. Synergistic antitumor effects of Rp1 with ActD were also observed in vivo. Our results suggest that combining Rp1 with chemotherapeutic agents could circumvent drug resistance and improve treatment efficacy.

## 1. Introduction

Chemotherapy is an effective way to manage many types of cancer; however, the prospects for a complete cure are limited by the emergence of primary and acquired drug resistance to chemotherapeutic agents [1]. Cancer cells develop multidrug resistance (MDR) through multiple mechanisms, including decreased drug uptake, increased drug efflux, activation of the DNA damage response, and enhanced cell survival [1]. For example, overexpression of ATP-binding cassette (ABC) transporter family proteins, such as P-glycoprotein (MDR1/ABCB1) and multidrug resistance protein 1 (MRP1/ABCC1), enhances efflux of anti-cancer drugs from cells [1,2]. Cancer cells can also acquire drug resistance by stimulating the repair of drug-induced DNA damage and inhibiting cell death signaling [3].

Sirtuin 1 (SIRT1) is a nicotinamide adenine dinucleotide-dependent enzyme that deacetylates various histone and non-histone substrates, such as p53, c-MYC, and FOXO, to regulate key biological processes, including DNA repair, cell cycle, apoptosis, cell survival, cellular metabolism, and cell senescence [4,5]. SIRT1 expression is upregulated in some cancers, such as prostate and colon cancer, and downregulated in others, such as breast cancer and hepatic cell carcinoma, suggesting that SIRT1 has different roles depending on cellular context [6]. Nonetheless, growing evidence implicates SIRT1 in cancer promotion and development of resistance to chemotherapeutical agents, including cisplatin, doxorubicin, and camptothecin [7,8,9]. Inhibition of SIRT1 decreases growth and viability of cancer cells while its overexpression impairs apoptosis, suggesting that SIRT1 is a critical regulator of cell proliferation and survival [7]. In addition, siRNA-mediated depletion of SIRT1 re-sensitizes cisplatin-resistant cancer cells to cisplatin [10]. Therefore, modulation of SIRT1 activity could be a viable strategy for overcoming MDR.

Ginsenosides, the active components of *Panax ginseng C.A. Meyer*, display anti-cancer properties [11,12] and reverse drug resistance. In mice implanted with adriamycin-resistant leukemia cells, ginsenosides Rg3 inhibit MDR1 activity by reducing membrane fluidity [13]. In adriamycin-resistant breast cancer cells, Rh2 attenuates adriamycin resistance by inhibiting MDR1 activity and increasing the rate and amount of adriamycin entering cellular/subcellular compartments [14]. We have previously reported that Rp1, a novel ginsenoside derivative, reverses MDR by downregulating MDR1 expression and inhibiting Src [2]. Aside from the effects on MDR1, the molecular mechanisms underlying ginsenoside modulation of chemosensitivity remain unclear. Actinomycin D (ActD) is an anti-cancer drug, which blocks DNA-dependent transcription and inhibits topoisomerase, resulting in DNA double stand breaks. Although ActD, an FDA-approved chemotherapeutic drug, has been used for treatment of various cancers, its use is limited by its toxicity at high dose [15,16]. For a beneficial use of ActD, combination therapy using a low dose should be exploited. Accordingly, in the present study, we intended to assess the anti-cancer effects of ginsenoside Rp1, ActD, and their co-administration in drug-resistant cells and murine xenograft model of colon cancer, and investigate the underlying mechanisms.

## 2. Results

### 2.1. Rp1 Enhances ActD-Induced Cell Death in Multidrug-Resistant LS513 Cells

**Figure 1:**
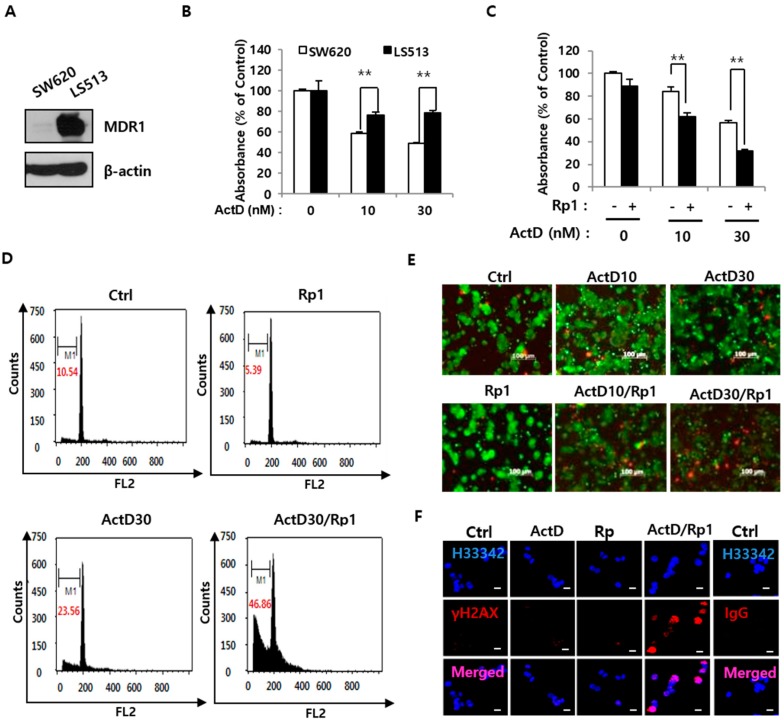
Synergic effect of Rp1 on cell death induced by ActD in the drug-resistant LS513 cells. (**A**) equal amount of total cellular proteins (20 μg) from SW620 cells and LS513 cells was processed for immunoblotting analysis using anti-MDR1 antibody. β-actin antibody was used as a loading control. (**B**) SW620 and LS513 cells were treated with indicated concentrations of actinomycin D (ActD) for 24 h and cell growth was measured by an MTS assay. Error bars represent standard deviation of the mean of three measurements (** *p* < 0.01). (**C**–**E**) LS513 cells were treated with indicated concentrations of ActD either in the presence or absence of 5 μM Rp1 for 24 h, followed by an MTS assay (** *p* < 0.01) (**C**), cell cycle analysis using flow cytometry (M1, sub-G1 phase) (**D**), and live-dead assay with cells stained with calcein AM (live, green) and PI (dead, red) (**E**). (**F**) LS513 cells were treated either with 5 μM Rp1, 30 nM ActD, or 5 μM Rp1 and 30 nM ActD together for 18 h, followed by immunofluorescence analysis using anti-γH2AX antibody. Isotype IgG was used as a control and Hoechst 33342 was used for nuclei stain. Scale Bar = 10 μM. These experiments were performed three times with comparable results.

We explored the effects of Rp1 and ActD on two human colon cancer cell lines, SW620 (drug-sensitive) and LS513 (multidrug-resistant). As MDR1 is expressed only in the latter cell line (Figure 1A), ActD was more cytotoxic in SW620 cells (Figure 1B). Previously, we have reported that Rp1 (5 μM) does not affect growth of drug-resistant ovarian cancer cells [2]. Similar to previous results, Rp1 (5 μM) alone had minimal effect on the growth of LS513 cells; however, in combination with ActD, it had a more potent inhibitory effect than did ActD alone (Figure 1C). Because 30 nM ActD showed better synergistic effects with Rp1 than 10 nM ActD (Figure 1C), we used 30 nM ActD in the further study. Cell cycle analysis revealed that co-administration of Rp1 and ActD increased the sub-G1 peak (hallmark of apoptosis), suggesting that the two agents act synergistically to promote cell death despite having minimal cytotoxic effects on their own (Figure 1D). In agreement with this, the live/dead staining revealed that co-administration of Rp1 and ActD yielded more dead cells than did treatment with Rp1 or ActD alone (Figure 1E). To gain insights into the mechanisms mediating this effect, we quantified the levels of histone γ-H2AX (indicator of DNA double-strand breaks [17]) to assess whether Rp1 could potentiate ActD-induced DNA damage. ActD (30 nM) did not affect γ-H2AX foci levels in LS513 cells, but Rp1 augmented ActD-induced γ-H2AX (Figure 1F). These data suggest that Rp1 enhances ActD-induced DNA damage in multidrug-resistant LS513 cells and, consequently, increases their sensitivity to ActD.

### 2.2. Rp1 Attenuates ActD-Induced SIRT1 Upregulation

**Figure 2:**
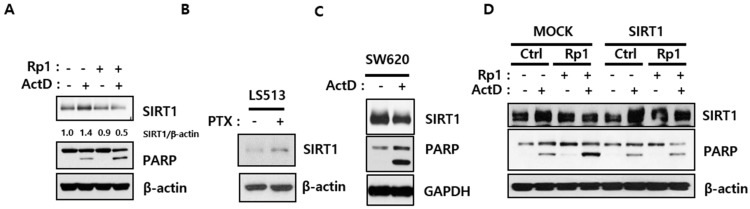
Correlation of decreased SIRT1 levels by Rp1 with sensitivity to ActD. (**A**,**B**) LS513 cells were treated either with 5 μM Rp1, 30 nM ActD, 5 μM Rp1, and 30 nM ActD together (**A**), or with 10 nM paclitaxel (PTX) (**B**) for 24 h, followed by immunoblotting analysis using indicated antibodies. (**C**) SW620 cells were treated with 30 nM ActD for 24 h, followed by immunoblotting analysis using indicated antibodies. A GAPDH antibody was used as a loading control; (**D**) LS513 cells transfected with either mock (empty vector) or SIRT1 plasmid were treated with 5 μM Rp1, 30 nM ActD or 5 μM Rp1 and 30 nM ActD for 24 h, followed by immunoblotting analysis using indicated antibodies. Similar results were observed in independent experiments.

Because overexpression of SIRT1 reportedly renders cancer cells resistant to anti-cancer drugs [18,19], we examined SIRT1 levels in multidrug-resistant LS513 cells. ActD upregulated SIRT1 expression while Rp1 attenuated this effect to enhance cell death, as determined by increased PARP cleavage (Figure 2A). Notably, ActD also upregulated SIRT1 levels in doxorubicin-resistant lung cancer cell line A549-DXR. Similar to ActD-treated LS513 cells, ActD-treated A549-DXR cells had higher SIRT1 levels and minimal PARP cleavage; concomitant administration of Rp1 and ActD re-sensitized the cells to ActD, as evidenced by decreased SIRT1 levels and increased PARP cleavage (Figure S1). Notably, paclitaxel was also able to simulate SIRT1 expression in LS513 cells (Figure 2B). Contrastingly, in ActD-sensitive SW620 cells, ActD decreased SIRT1 levels and increased PARP cleavage (Figure 2C). These results suggest that reduced SIRT1 levels are important for chemosensitivity of cancer cells. To further explore the notion that Rp1 re-sensitizes L513 cells to ActD by downregulating SIRT1, we overexpressed SIRT1 in LS513 cells. SIRT1 overexpression attenuated PARP cleavage induced by Rp1 and ActD co-treatment (Figure 2D). Collectively, these data imply that SIRT1 plays a critical role in drug resistance and that Rp1 could reverse drug resistance by downregulating SIRT1.

### 2.3. SIRT1 Inhibition Reverses Resistance to ActD through p53 Deacetylation

**Figure 3:**
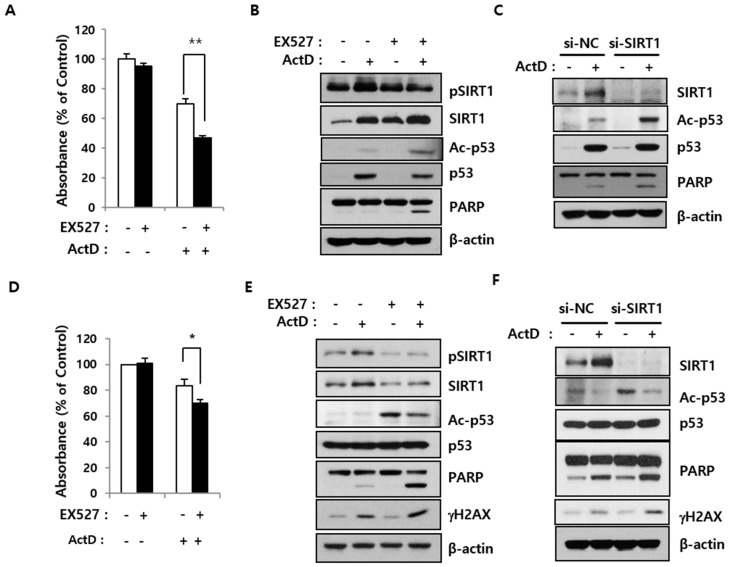
Effects of SIRT1 inhibition on ActD-induced cell death. (**A**,**B**,**D**,**E**) LS513 cells (**A**,**B**) were treated either with 30 nM ActD, 50 μM EX527, or 30 nM ActD and 50 μM EX527 together and OVCAR-DXR cells (**D**,**E**) were treated either with 1 μM ActD, 50 μM EX527, or 1 μM ActD and 50 μM EX527 together for 24 h. Cells were then subjected to either MTS assay (**A**,**D**) or immunoblotting analysis using indicated antibodies (**B**,**E**). (* *p* < 0.05, ** *p* < 0.01) (**C**,**F**) LS513 cells (**C**) and OVCAR-DXR cells (**F**) were transfected either with si-NC or si-SIRT1 RNA for 24 h and then treated with 30 nM or 1 μM ActD for 24 h, respectively. Cell lysates were subjected to immunoblotting analysis using indicated antibodies. The experiments were performed with similar results independently.

To further investigate whether SIRT1 activity is important for ActD resistance, cells were treated with a selective SIRT1 inhibitor, EX527. While EX527 (50 μM) alone was only mildly cytotoxic, in combination with ActD, it significantly impaired the growth of both LS513 and OVCAR-DXR cells (multidrug-resistant cells derived from the human ovarian cancer cell line OVCAR-8 [2]) (Figure 3A,D). ActD treatment increased the levels of total and phosphorylated SIRT1 (the active form of SIRT1 [20]), while EX527 had the opposite effect. SIRT1 deacetylates p53 to decrease cell death [21]. Accordingly, co-exposure to EX527 and ActD promoted p53 acetylation and synergistically induced cell death, as evidenced by increased PARP cleavage (Figure 3B,E). Next, we tested whether siRNA-mediated silencing of SIRT1 could re-sensitize drug-resistant cells to ActD. SIRT1 knockdown abrogated ActD-induced SIRT1 upregulation to increase p53 acetylation and PARP cleavage in LS513 and OVCAR-DXR cells (Figure 3C,F). However, SIRT1 inhibition by itself, either pharmacological or siRNA-mediated, was insufficient to induce cell death even though p53 acetylation was markedly stimulated in OVCAR-DXR cells (Figure 3E,F). SIRT1 inhibition in combination with ActD treatment synergistically enhanced cell death and DNA damage, as determined by increased γ-H2AX levels (Figure 3E,F). Taken together, these results suggest that ActD upregulates SIRT1, which is responsible for the development of drug resistance.

**Figure 4:**
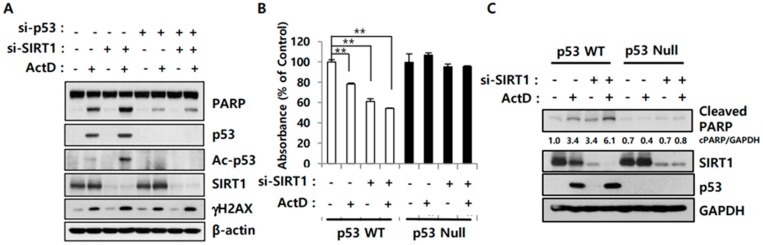
Importance of p53 in SIRT1-regulated cell death induced by ActD. (**A**) LS513 cells were transfected either with si-NC, si-SIRT1, si-p53, or si-SIRT1 and si-p53 together for 24 h, and then were treated with 30 nM ActD for 24 h. Cell lysates were subjected to immunoblotting analysis using indicated antibodies. (**B**,**C**) HCT116-p53 WT and HCT116-p53 null cells were transfected either with si-NC or si-SIRT1 for 24 h, and then treated with 30 nM ActD for 24 h. Cell growth was measured by MTS assay (**B**) (** *p* < 0.05) and cell lysates were subjected to immunoblotting analysis using indicated antibodies. (**C**) Similar results were observed in independent experiments.

ActD treatment upregulated p53 expression, but the levels of acetylated p53 were minimal, probably due to SIRT1 induction. Inhibition of SIRT1 enhanced p53 acetylation and ActD-induced cell death (Figure 3). To further evaluate the role of p53 in SIRT1 inhibition-mediated drug sensitivity, we depleted p53 expression using siRNA. Although si-SIRT1 treatment enhanced ActD-induced PARP cleavage in LS513 cells, this synergistic effect was reduced when p53 was knocked down despite comparable γ-H2AX levels (Figure 4A). To verify whether p53 mediates the synergistic effect of SIRT1-knockdown and ActD, we assessed the growth of p53 wild-type (WT) and p53-null human colon cancer HCT116 cells [22]. SIRT1 knockdown enhanced ActD-induced apoptosis of HCT116-p53 WT cells, but not of HCT116-p53-null cells, as determined by the MTS assay (Figure 4B) and PARP cleavage (Figure 4C). These data suggest that p53 expression is necessary for SIRT1 regulation of drug sensitivity.

### 2.4. Activation of AKT Correlates with SIRT1 Upregulation in Drug-Resistant Cells

**Figure 5:**
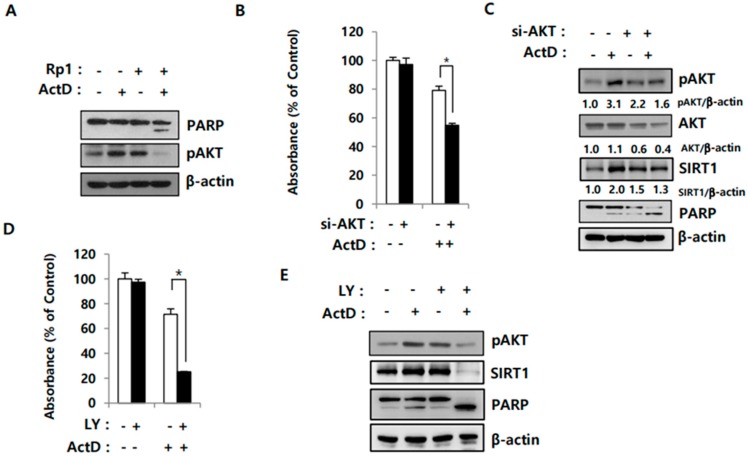
AKT regulation of SIRT1 and cell death induced by ActD. (**A**) LS513 were treated either with 5 μM Rp1, 30 nM ActD, or 5 μM Rp1 and 30 nM ActD together for 24 h, followed by immunoblotting analysis using indicated antibodies. (**B**,**C**) LS513 cells transfected either with si-NC or si-AKT for 24 h and cells were treated with 30 nM ActD for 24 h, followed by MTS assay (**B**) and immunoblotting analysis using indicated antibodies (**C**). (**D**,**E**) LS513 cells were treated either with 30 nM ActD, 20 μM LY294002 (LY) or 30 nM ActD and 20 μM LY for 24 h, followed by MTS assay (**D**) and immunoblotting analysis using indicated antibodies (**E**). (* *p* < 0.05) Similar results were observed in independent experiments.

The PI3K/AKT pathway is associated with MDR in various cancer cells, such as doxorubicin-resistant breast cancer and fluorouracil-resistant colon cancer cells [23,24]. SIRT1 promotes membrane localization and activation of AKT [25]. Therefore, we examined whether the synergistic effects of Rp1 and ActD are mediated through the SIRT1-AKT pathway. ActD enhanced AKT activity, as evidenced by increased levels of phosphorylated AKT; Rp1 alone also stimulated AKT activity, although to a lesser extent than ActD (Figure 5A). When AKT was knocked down, cell death was significantly increased upon ActD treatment, as determined by the MTS assay (Figure 5B) and PARP cleavage (Figure 5C). AKT knockdown by itself had minimal effect on SIRT1 in control cells, but it significantly downregulated SIRT1 expression in ActD-treated cells (Figure 5C), suggesting that SIRT1 could be regulated by AKT. To explore this possibility, cells were treated with LY294002, a PI3K/AKT inhibitor. As expected, LY294002 significantly reduced cell viability and abolished ActD-induced increase in SIRT1 expression in ActD-treated cells (Figure 5D,E). These findings imply that ActD-induced activation of AKT upregulates SIRT1, which contributes to the development of drug resistance.

### 2.5. Rp1 Attenuates Resistance to ActD through the Cholesterol-Associated AKT/SIRT1 Pathway

**Figure 6:**
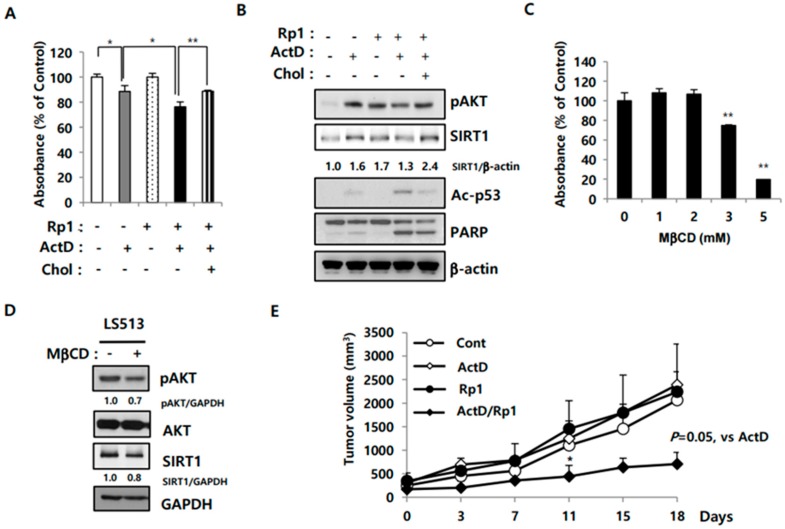
Effect of cholesterol on SIRT1-regulated cell death induced by ActD. (**A**,**B**) LS513 cells were treated either with 30 nM ActD, 5 μM Rp1, or 30 nM ActD and 5 μM Rp1 together for 24 h. For cholesterol treatment (Chol), cells were pretreated with 200 μM cholesterol for 4 h. Cell growth inhibition was measured by an MTS assay (**A**) and cell lysates were processed for immunoblotting analysis using indicated antibodies (**B**) (* *p* < 0.05, ** *p* < 0.01); (**C**) LS513 cells were treated with the indicated concentrations of methyl-beta cyclodextrin (MβCD) for 24 h, and cell growth inhibition was assessed by an MTS assay. (** *p* < 0.01) (**D**) LS513 cells were treated with 2.5 mM MβCD, and then cell lysates were subjected to immunoblotting analysis using indicated antibodies. (**E**) LS513 (3 × 10^6^) cells were injected subcutaneously into nude mice. When the tumor reached an average volume of 100 mm^3^, the mice were assigned randomly to four groups (*n* = 3 mice per group). Mice were treated either with 0.03 mg/kg ActD, 5 mg/kg Rp1, or 0.03 mg/kg ActD and 5 mg/kg Rp1 together intraperitoneally twice per week. The control group was injected with a vehicle (PBS). Tumor size was measured at the indicated times with error bars representing standard deviation. (* *p* < 0.05). The experiments were performed with similar results independently.

Previously, we reported that Rp1 modulates lipid rafts- cholesterol-enriched membrane microdomains where AKT is recruited and activated [26]. Cholesterol is a critical structural and functional component of lipid rafts; its depletion causes lipid raft disruption and cell death through AKT inactivation, which can be rescued with cholesterol addition [27,28]. Therefore, we tested whether cholesterol addition could attenuate Rp1-induced drug sensitivity. Cholesterol treatment reversed the synergistic inhibition of cell growth (Figure 6A) and recovered both AKT activity and SIRT1 upregulation in Rp1 and ActD co-treated cells, leading to decreased p53 acetylation and PARP cleavage (Figure 6B). To explore the mechanisms mediating the cytotoxic effects of cholesterol depletion, cholesterol was depleted from LS513 cells using methyl-β-cyclodextrin (MβCD). MβCD treatment effectively impaired the growth of LS513 cells (Figure 6C), downregulated SIRT1 expression, and inhibited AKT inactivation (Figure 6D). Collectively, these results suggest that Rp1 and ActD act synergistically through downregulation of the AKT/SIRT1 pathway, which can be modulated by cholesterol levels and/or lipid raft integrity. To evaluate the anti-cancer effects of Rp1 and ActD co-treatment in vivo, xenografts were generated in nude mice through subcutaneous injection of LS513 cells. While ActD and Rp1 had a limited effect on their own, they effectively hindered tumor growth when used in combination (Figure 6E).

**Figure 7:**
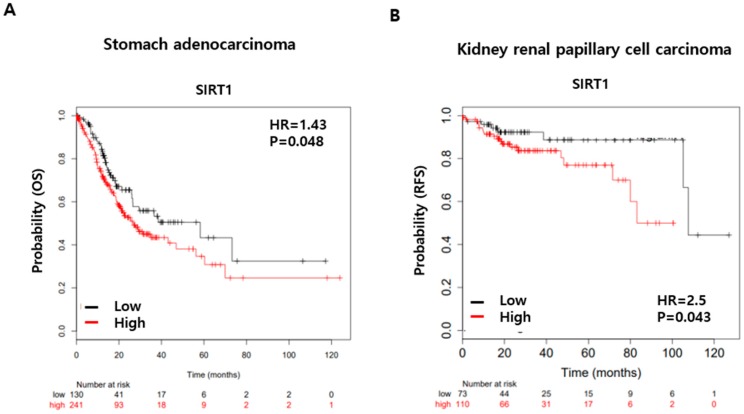
Kaplan–Meier survival analysis from publicly available data. (**A**,**B**) Kaplan–Meier survival curves relative to expressions of SIRT1 Kaplan–Meier overall survival (OS) curves for stomach adenocarcinoma (**A**), and recurrence free survival (RFS) for kidney renal papillary cell carcinoma patients (**B**) with high (red) and low (black) expression of SIRT1 gene from the KM plotter database. The hazard ratio (HR) and log-rank *p*-value are in each figure.

To evaluate the prognostic value of SIRT1 expression in cancer, we used the publicly available Kaplan–Meier Plotter database (http://kmplot.com) to download gene expression and survival data of various cancer patients. As shown in Figure 7A,B, SIRT1 expression was significantly correlated with poor overall survival (OS) in stomach adenocarcinoma patients and poor recurrence-free survival (RFS) in kidney renal papillary cell carcinoma patients (*p* < 0.05). Albeit not statistically significant, a negative correlation between SIRT1 expression and OS was also observed in esophageal squamous cell carcinoma, kidney renal papillary cell carcinoma, lung squamous cell carcinoma, sarcoma, and uterine corpus endometrial carcinoma patients; a negative association between SIRT1 expression and RFS was also noted in cervical squamous cell carcinoma, esophageal squamous cell carcinoma, head-neck squamous cell carcinoma, kidney renal papillary cell carcinoma, pancreatic ductal adenocarcinoma, and stomach adenocarcinoma patients (Figure S2A,B). Taken together, these results suggest that SIRT1 overexpression is associated with ActD resistance, and that inhibiting SIRT1 with Rp1 could be of therapeutic benefit to overcome drug resistance in tumor cells.

## 3. Discussion

Although conventional chemotherapy is widely used for the treatment of cancer, anti-cancer agents are often toxic not only to tumor cells but also to healthy cells [29]. Worse still, cancer cells can transition from a chemotherapy-sensitive to a chemotherapy-resistant phenotype, which is a major cause of treatment failure and metastasis [3]. Thus, it is very important to explore novel strategies to overcome MDR. Several mechanisms are involved in drug resistance, including enhanced efflux of chemotherapeutic agents from cancer cells, activation of cell survival signaling, and inhibition of apoptosis. This study provides in vitro and in vivo evidence for synergistic inhibitory action of ginsenoside Rp1 with ActD on the growth of drug-resistant cancer cells. We demonstrated that these effects are exerted through inactivation of the AKT/SIRT1 pathway and increase in p53 acetylation.

Natural compounds with anticancer properties have generated considerable interest among researchers owing to their safety and efficacy [29]. Ginsenosides, the main active compounds extracted from *P. ginseng*, display a wide range of therapeutic and pharmacological activities, including antioxidant, anti-inflammatory, and anti-cancer effects [30]. Rg3, one of the active ginsenosides, exerts antitumor effects in various cancers through induction of apoptosis and inhibition of proliferation, metastasis, angiogenesis, and MDR [31]. We previously reported that Rp1 inhibits MDR through downregulation of MDR1 and modulation of lipid rafts [2]. In this study, we further elucidated the mechanisms of Rp1 modulation of drug sensitivity, focusing on the AKT-SIRT1-p53 pathway.

Recently, an association between SIRT1 expression and drug resistance has been reported [4,10]. SIRT1 regulates various biological functions, such as cell proliferation, cell survival, immune response, and carcinogenesis. Although the roles of SIRT1 in cancer are controversial, its overexpression is a feature of many solid tumors and hematopoietic malignances [32,33,34]. Our analysis of publicly available Kaplan–Meier data revealed that SIRT1 expression is negatively correlated with survival in several human cancer types (Figure 7). Moreover, accumulating evidence suggests that SIRT1 is activated in multidrug-resistant cell lines. SIRT1 positively regulates the expression of ABC transporters, such as MDR1 and ABCA1, which promote efflux of anti-cancer drugs from cells [35]. We observed that, upon ActD treatment, SIRT1 was upregulated in drug-resistant LS513, OVCAR-DXR, and A549-DXR cells, and downregulated in drug-sensitive SW620 cells (Figure 2, Figure 3, and Figure S1). SIRT1 upregulation is not limited to ActD-treated cells, as paclitaxel caused a similar effect in LS513 cells (Figure 2B). In addition, SIRT1 inhibition, either pharmacological or through gene knockdown, augmented ActD-induced cell growth inhibition and PARP cleavage, suggesting that SIRT1 upregulation is involved in resistance to ActD (Figure 3). Therefore, it is interesting that Rp1 in combination with ActD, but not Rp1 alone, downregulated SIRT1 (Figure 2A). When SIRT1 was overexpressed, Rp1 was unable to re-sensitize cells to ActD, suggesting that upregulation of SIRT1 plays an important role in drug resistance (Figure 2D). SIRT1-mediated drug resistance appeared not to be related to MDR1 expression because SIRT1 knockdown had a limited effect on MDR1 expression in LS513 cells.

Certain chemotherapeutic agents, including ActD, exert their cytotoxic effects by damaging the DNA. Cancer cells are able to boost alternative DNA repair pathways to avoid apoptosis, thus developing drug resistance [36]. SIRT1 deacetylates several master transcriptional factors involved in apoptosis and DNA damage, including p53, to inhibit apoptosis [21]. SIRT1-deficient cells exhibit p53 hyperacetylation following DNA damage [37]. In LS513 cells, although ActD could upregulate p53 expression, p53 acetylation was minimal, probably due to ActD-induced SIRT1 upregulation (Figure 3B,C). When SIRT1 was inhibited, either by EX527 or siRNA-mediated silencing, p53 acetylation and PARP cleavage increased upon ActD treatment (Figure 3B,C). Rp1 attenuated ActD-induced SIRT1 upregulation to increase p53 acetylation, leading to synergistic anti-cancer effects in drug-resistant cells (Figure 6B).

Bhatia et al. reported that pharmacological inhibition of SIRT1 or SIRT1 knockdown increase apoptosis in leukemia stem cells, and that the inhibitory effects of SIRT1 depend on p53 expression and acetylation [38]. SIRT1 can prevent oxidative stress-induced apoptosis in mesangial cells through p53 deacetylation [39]. Thus, we investigated whether the SIRT1-p53 deacetylation pathway is important for ActD-mediated cell death. ActD treatment enhanced PARP cleavage and p53 acetylation in SIRT1-deficient LS513 cells (Figure 4A). However, ActD could not increase PARP cleavage when both SIRT1 and p53 were knocked down even though ActD-induced DNA damage (assessed by γ-H2AX levels) was similar to that in SIRT1-depleted cells (Figure 4A). In line with these results, SIRT1 knockdown re-sensitized p53-expressing HCT-116 cells, but not p53-deficient HCT-116 cells, to ActD (Figure 4B,C), implying that p53 activation through SIRT1 inhibition is critical for ActD-induced cell death.

The PI3K/AKT pathway is often activated in cancer and contributes to tumorigenesis, metastasis, and chemoresistance [40,41]. Recently, PI3K/AKT signaling has been reported to modulate chemoresistance by regulating ABGG2 expression in human multiple myeloma [42]. In our study, ActD promoted SIRT1 upregulation as well as AKT phosphorylation (Figure 5C). It is possible that SIRT1 upregulation contributes to ActD-induced AKT activation because SIRT1 is known to deacetylate AKT. Deacetylation of AKT is necessary for its binding to PIP3 and, in turn, its membrane localization and activation [25]. However, SIRT1 knockdown decreased AKT phosphorylation only to a small extent. AKT inactivation, either through siRNA or using LY294002, a PI3K/AKT inhibitor, deceased SIRT1 levels and re-sensitized LS513 cells to ActD (Figure 5B–E), implying that SRIT1 could be a downstream target of AKT.

An intact structure of lipid rafts, cholesterol-enriched membrane microdomains, is critical for AKT activation and, thus, cell survival [28]. Exposure to ActD stimulated AKT activity and SIRT1 levels, which was attenuated by addition of Rp1 (Figure 6B). Rp1 (5 μM) alone had a minimal effect on cell viability, but when combined with 30 nM ActD, the two agents acted synergistically to induce cell death through AKT inactivation and downregulation of SIRT1 (Figure 6B). It is possible that co-treatment with Rp1 and ActD amplifies modification of lipid rafts, leading to AKT inactivation and drug sensitivity. In support of this notion, treatment with a lipid-raft-disrupting agent, MβCD, caused AKT inactivation and SIRT1 downregulation in LS513 cells (Figure 6C). Cholesterol addition, which is known to fortify lipid rafts and increase AKT activation [28], could preserve SIRT1 levels and thus reverse drug sensitivity induced by Rp1 and ActD co-treatment (Figure 6), implicating lipid rafts in Rp1-medated drug sensitivity.

## 4. Materials and Methods

### 4.1. Materials

Ginsenoside Rp1 (purity, 99%), a gift from Ambo Institute (Seoul, Korea), was dissolved in dimethyl sulfoxide (DMSO; Sigma-Aldrich, St. Louis, MO, USA) [43]. ActD was purchased from Sigma-Aldrich. Anti-MDR1, anti-SIRT1, normal rabbit and mouse IgG, HRP-conjugated rabbit, and mouse IgG antibodies were purchased from Santa Cruz Biotechnology (Santa Cruz, CA, USA). Anti-phospho-H2AX (Ser139, γ-H2AX) antibody was obtained from Millipore Corporation (Bedford, MA, USA). Antibodies against PARP, acetyl p53, phospho-SIRT1, phospho-AKT, and AKT were purchased from Cell Signaling Technology (Beverly, MA, USA). Anti-β-actin antibody was obtained from Sigma-Aldrich.

### 4.2. Cell Culture

Human colorectal cancer cell lines, LS513 (drug resistant) and SW620 (lymph node metastasis) were obtained from the Korean Cell Line Bank (KCLB, Seoul, Korea) and the American Type Culture Collection (ATCC, Rockville, MD, USA), respectively. Human colon cancer cells, HCT116 p53 WT (wild type p53) and HCT116 p53-null (p53 null mutant) are a kind gift from Dr. Sung-Ho Goh (National Cancer Center, Goyang, Korea).

HCT116 p53WT, HCT116 p53-null, SW620, and LS513 cells were grown in RPMI 1640 medium with L-glutamine (Hyclone, Logan, UT, USA) supplemented with 10% FBS (Hyclone), 100 U/mL penicillin, and 100 µg/mL streptomycin (Antibiotics-Antimycotic, Gibco Laboratories Co., Grand Island, NY, USA) as previously described [2]. OVCAR-DXR cells were grown under selective pressure of 1 µM doxorubicin. The cells were allowed to adhere overnight and reach approximately 70% confluence. Before treatment, the cells were serum starved for 4 h using RPMI 1640 medium with 0.1% bovine serum albumin (BSA; USB Corp., Cleveland, OH, USA).

### 4.3. Cell Viability and Proliferation Assays

For the cell viability assay, cells were exposed to indicated concentrations of reagents in RPMI 1640 medium containing 0.1% BSA. Following incubation, the cells were stained with green-fluorescent calcein AM and red-fluorescent ethidium homodimer-1. For the cell proliferation assay, LS513 cells (5 × 10^4^ cells/well) were plated in a 96-well culture plate for 24 h before treatment (approximately 70% confluence). Cell growth was determined using the CellTiter 96 Kit (MTS, 3-(4, 5-dimethylthiazol-2-yl)-5-(3-carboxyme-thoxyphenyl)-2-(4-sulfophenyl)-2H-tetrazolium; Promega, Madison, WI, USA) as previously described [2].

### 4.4. siRNA Transfection

SIRT1 plasmid was kindly provided by Dr. Dong Hoon Shin (National Cancer Center, Korea). Reverse transfection of siRNA duplexes into cells was performed using Lipofectamine RNAiMAX (Invitrogen, Carlsbad, CA, USA) while transfection of plasmids into cells was performed using Lipofectamine 2000 (Invitrogen) according to the manufacturer’s instructions. The sequences of siRNA for the negative control (NC), SIRT1, and AKT were 5′-CCUACGCCACCAAUUUCGU-3′ (NC, Bioneer, Daejeon, Korea) and 5′-CUAAUCUAGACCAAAGAAU-3′ (SIRT1, Bioneer) 5′-GACAACCGCCAUCCAGACU (AKT, Bioneer), respectively.

### 4.5. Immunoblotting Analysis

Cells were lysed with 2 x SDS lysis buffer (20 mM Tris, pH 8.0, 2 mM EDTA, 1 mM Na_3_VO_4_, 1 mM DTT, 2% SDS, 20% glycerol) and boiled for 5 min, followed by protein assay to determine protein concentration of each sample using Micro BCA Protein Assay Reagent (Pierce, Rockford, IL, USA). Total cellular protein (20 μg) was separated by 8 or 12% SDS-PAGE and transferred to polyvinylidene difluoride membranes. The membranes were blocked at room temperature (RT) in tris-buffered saline and tween 20 (TBS-T) containing 5% non-fat dried milk. The membranes were incubated with the primary antibody overnight at 4 ˚C, washed two times with TBS-T for 30 min, incubated with HRP-conjugated goat anti-mouse IgG or goat anti-rabbit IgG secondary antibodies for 1 h at RT, and then washed with TBS-T two times for 15 min. The labeled proteins were visualized by the enhanced chemi-luminescence method. The levels of protein were quantified by a densitometry and normalized to loading control β-actin or GAPDH. Densitometry readings maybe found in Supplemental data (Figure S3).

### 4.6. Immunofluorescence and Confocal Microscopy Imaging

Cells were grown on to glass cover slips and fixed with 2% paraformaldehyde in PBS. The fixed cells were rinsed with PBS, permeabilized with 0.2% Triton X-100 in PBS for 5 min, and then blocked with 3% BSA in PBS for 1 h at room temperature. Subsequently, the cells were incubated with indicated primary antibodies or non-specific IgG overnight at 4 ˚C, washed in PBS, and then exposed to Alexa 568-conjugated secondary IgG for 1 h at room temperature, respectively. The cells were stained with Hoechst 33342 before the glass cover slips were washed in PBS and mounted on glass slides. The cells were examined under the Zeiss LSM 510-Meta confocal fluorescence microscope (Carl Zeiss, Jena, Germany).

### 4.7. Flow Cytometric Analysis of Cell Death

Cells were harvested, fixed with 70% ethanol, and stained with propidium iodide (PI) solution (20 µg/mL PI, 0.1% sodium citrate, 50 µg/mL RNase A, 0.03% NP-40, PBS) before they were analyzed by flow cytometry with CellQuest software (BD Biosciences, San Jose, CA, USA).

### 4.8. Establishment of a Murine Xenograft Model

LS513 cells (3 × 10^6^) were suspended in PBS, mixed with Matrigel (1:1, *v/v*; BD Biosciences, Bedford, MA, USA), and then injected subcutaneously into 6-week-old Balb/c nude mice (0.1 mL per animal). Tumor length (L) and width (W) were measured using a caliper, and the average tumor volume was calculated as (L × W^2^)/2. When the tumors reached an average volume of ~100 mm^3^, the mice were randomized into four groups, with 3 mice per group. The mice were treated by intraperitoneal injection twice a week either with PBS (as a control), ActD, Rp1, or both ActD and Rp1. The mice in both control group (PBS-treated) and drug-treated group were fed with a standard chow diet (altromin 1314; Altromin, Lage, Germany). Tumor size was measured with calipers. This study was reviewed and approved by the Institutional Animal Care and Use Committee (IACUC) of National Cancer Center Research Institute (NCCRI). NCCRI is an Association for Assessment and Accreditation of Laboratory Animal Care International (AAALAC International) accredited facility and abides by the Institute of Laboratory Animal Resources (ILAR) guide.

## 5. Conclusions

In the present study, ActD treatment upregulated SIRT1 levels and activated AKT in multidrug-resistant cells. We showed that inhibition and/or downregulation of either SIRT1 or AKT could re-sensitize the cells to ActD, and that Rp1 downregulated both SIRT1 and AKT when co-administered with ActD. Taken together, our results suggest that ActD in combination with Rp1 may be an effective chemotherapeutic strategy for overcoming drug resistance of cancer cells.

## Supplementary Materials

The following are available online at https://www.mdpi.com/2072-6694/12/3/605/s1, Figure S1: Synergic effect of Rp1 on cell death induced by ActD in A549/DXR cells, Figure S2: Kaplan–Meier survival analysis from publicly available data, Figure S3: Original Western blots.
