# Supplementary material for: Ginsenoside Rp1, A Ginsenoside Derivative, Augments Anti-Cancer Effects of Actinomycin D via Downregulation of an AKT-SIRT1 Pathway
Source: Cancers (Basel). 2020 Mar 5;12(3):605. doi: 10.3390/cancers12030605 (PMC7139315; doi:10.3390/cancers12030605)
Supplement: Supplementary file 1 [file cancers-12-00605-s001.pdf]

Supplementary Materials

Ginsenoside Rp1, A Ginsenoside Derivative, Augments Anti-Cancer Effects of Actinomycin D via Downregulation of AKT-SIRT1 Pathway

Un-Jung Yun, In Hye Lee, Jae-Seon Lee, Jaegal Shim and Yong-Nyun Kim

Materials and Methods

1. Materials

Anti-SIRT1, anti-PARP, antibodies were purchased from Cell Signaling Technology (Beverly, MA). Anti- -actin antibody was purchased from Sigma-Aldrich Corporation (St. Louis, MO).

2. Cell Culture

Human lung cancer cell line A549 was obtained from the American Type Culture Collection (ATCC, Rockville, MD) and established by stepwise exposures to increasing concentrations of doxorubicin (A549-DXR). A549-DXR was grown in RPMI with L-glutamine (Hyclone, Logan, UT) supplemented with 10% FBS, 100 units/ml penicillin, 100 g/ml streptomycin, (Gibco Laboratories Co., Grand Island, NY) at 37°C in a humidified atmosphere containing 5% CO_2. Cells were allowed to adhere overnight and grown to approximately 70% confluence, and were serum starved for 4 hr using RPMI containing 0.1% bovine serum albumin (BSA, USB Corp., Cleveland, OH) prior to treatment. Cells were treated with indicated concentrations of reagents in the RPMI containing 0.1% BSA

3. Immunoblotting Analysis

Cells were lysed with 2 x SDS lysis buffer (20 mM Tris, pH 8.0, 2 mM EDTA, 1 mM Na_3VO_4, 1 mM DTT, 2% SDS, 20% glycerol) and boiled for 5 min, followed by protein assay to determine protein concentration of each sample using Micro BCA Protein Assay Reagent (Pierce, Rockford, IL). Total cellular protein (20 μg) was separated by 8 or 12% SDS-PAGE and transferred to polyvinylidene difluoride membranes. The membranes were blocked at room temperature (RT) in tris-buffered saline and tween 20 (TBS-T) containing 5% non-fat dried milk. The membranes were incubated with the primary antibody overnight at 4℃, washed two times with TBS-T for 30 min, incubated with HRP-conjugated goat anti-mouse IgG or goat anti-rabbit IgG secondary antibodies for 1 hr at RT, and then washed with TBS-T two times for 15 min. The labeled proteins were visualized by the enhanced chemi-luminescence method. The levels of protein were quantified by a densitometry and normalized to loading control β-actin or GAPDH.

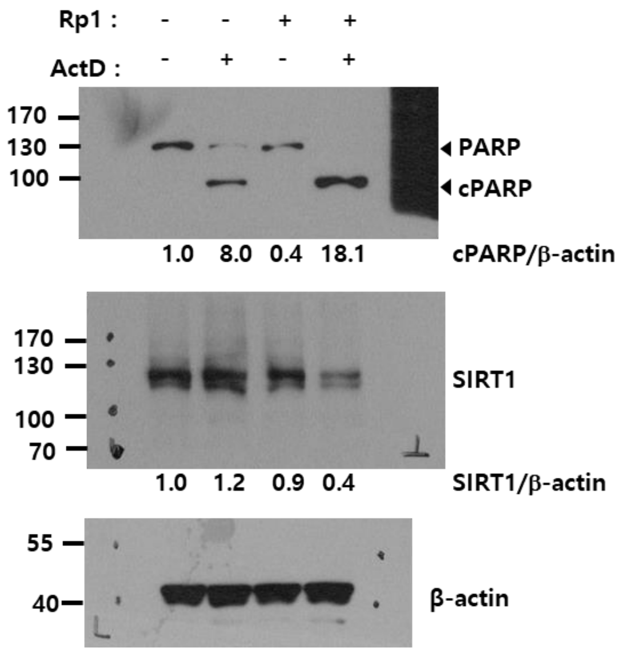

Figure S1. Synergic effect of Rp1 on cell death induced by ActD in A549/DXR cells. A549-DXR were treated with 5 μM Rp1, 50 nM actinomycin D (ActD) or 5 μM Rp1 and 50 nM ActD for 24 h and followed by immunoblotting analysis using anti-SIRT1, anti-PARP, and –actin antibody was used as a loading control. Similar results were observed in two independent experiments. Cleaved PARP (cPARP).

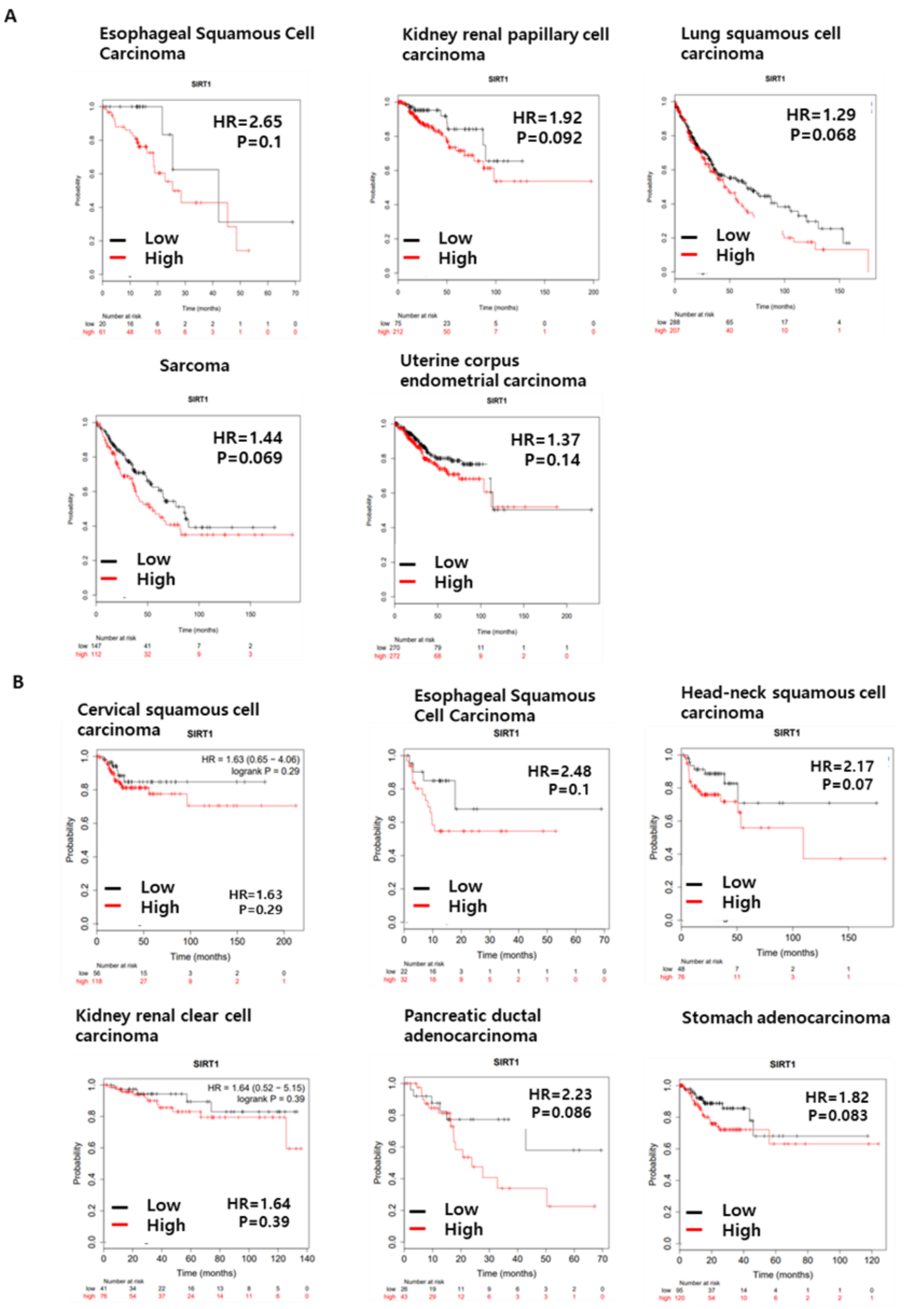

Figure S2. Kaplan-Meier survival analysis from publicly available data. (**A**,**B**) Kaplan-Meier curve showed probability for Overall Survival (**A**) and Recurrence Free Survival (RFS) (**B**) of patients with various cancer expressing high or low level of SIRT1.

Fig 1A

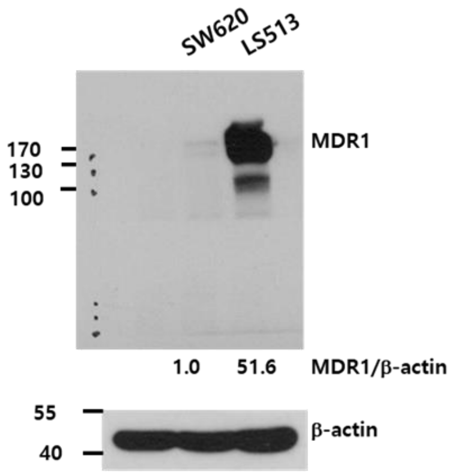

Fig 2A

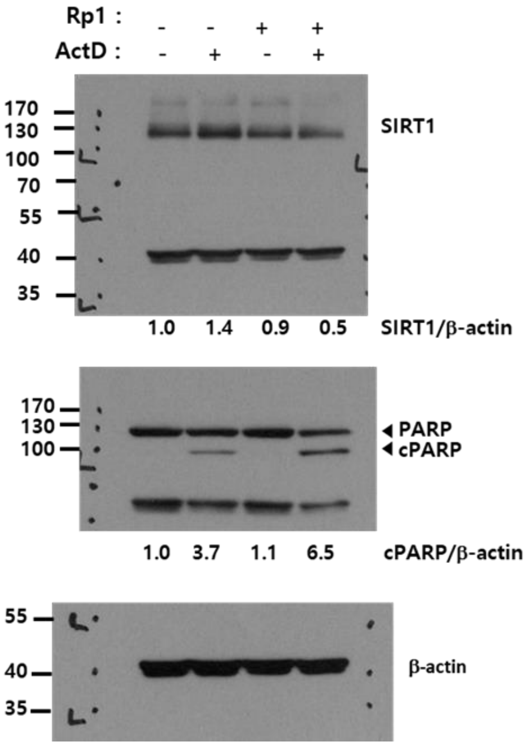

Fig 2B

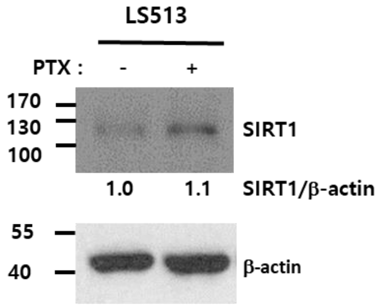

Fig 2C

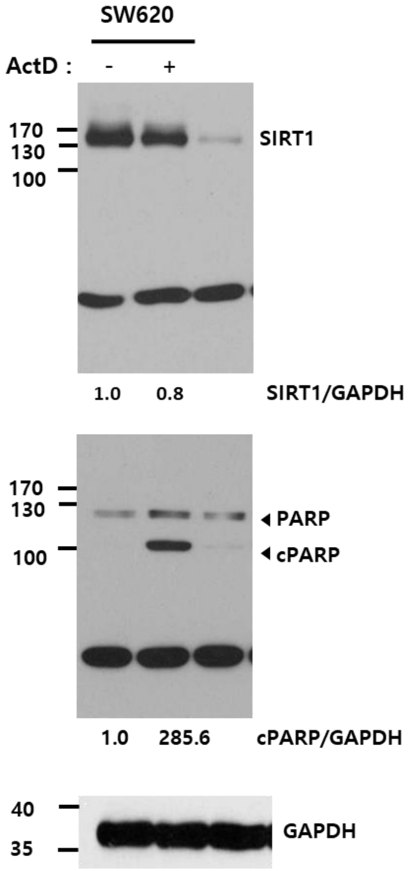

Fig 2D

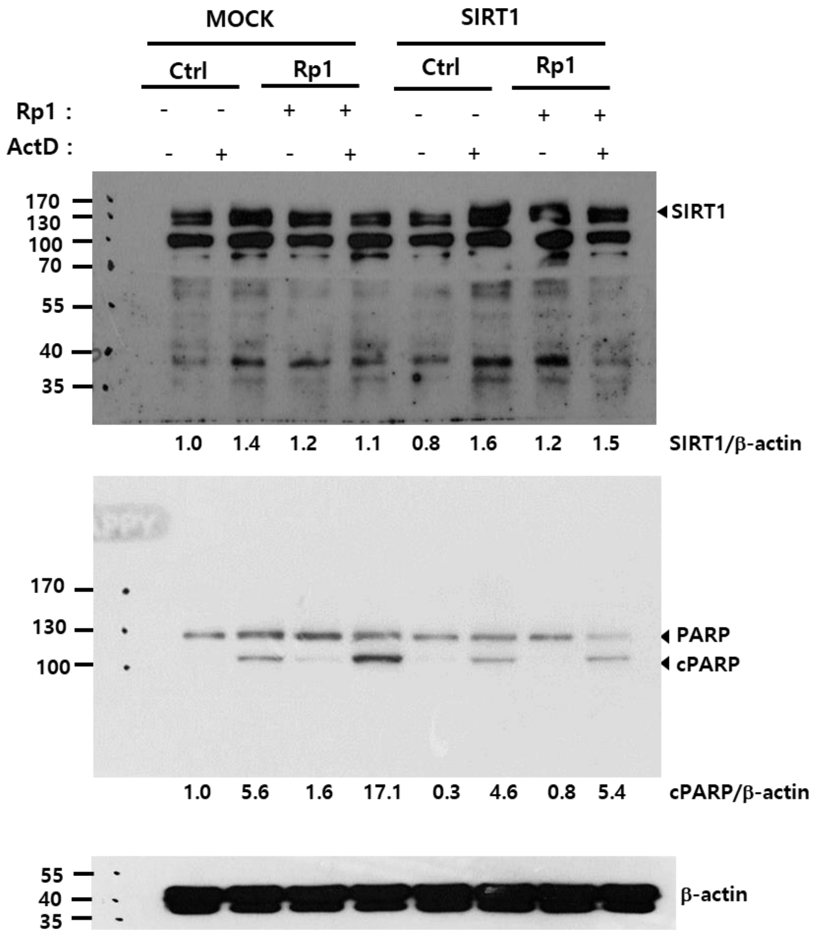
MOCK
SIRT1
Ctrl
Rp1
Ctrl
Rp1
Rp1 : - - + + - - + +
ActD : - + - + - + - +
170
130
100
70
55
40
35
SIRT1
1.0 1.4 1.2 1.1 0.8 1.6 1.2 1.5
SIRT1/β-actin
170
130
100
PARP
cPARP
1.0 5.6 1.6 17.1 0.3 4.6 0.8 5.4
cPARP/β-actin
55
40
35
β-actin

Fig 3B

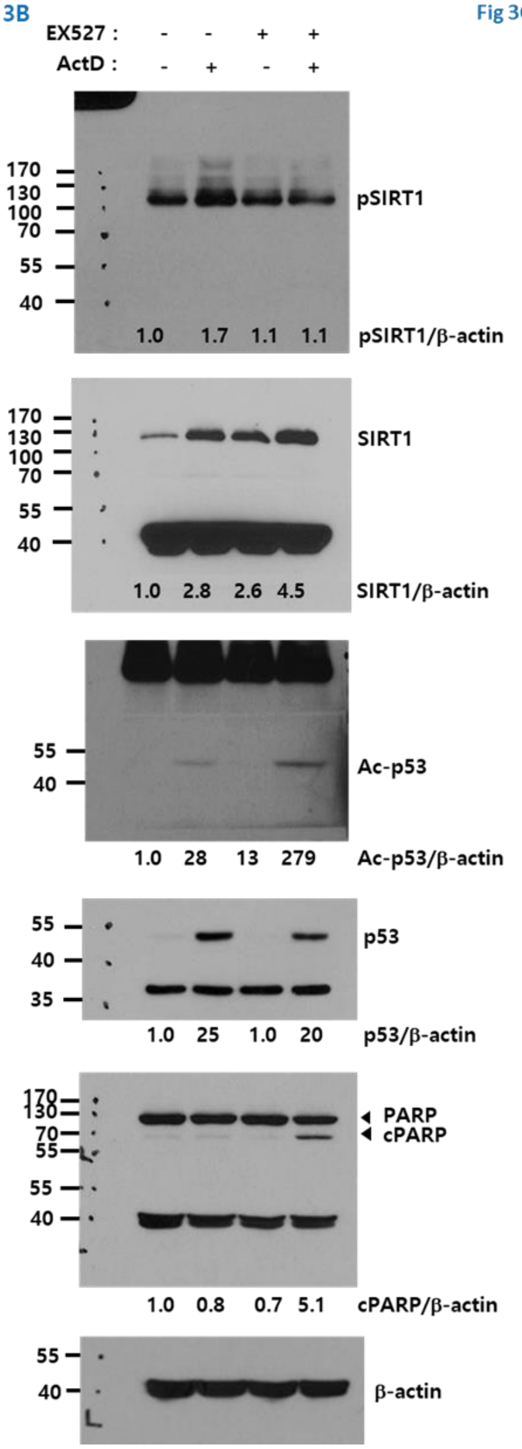
EX527 : - - + +
ActD : - + - +
170
130
100
70
55
40
pSIRT1
1.0 1.7 1.1 1.1
pSIRT1/β-actin
170
130
100
70
55
40
SIRT1
1.0 2.8 2.6 4.5
SIRT1/β-actin
55
40
Ac-p53
1.0 28 13 279
Ac-p53/β-actin
55
40
35
p53
1.0 25 1.0 20
p53/β-actin
170
130
70
55
55
40
PARP
cPARP
1.0 0.8 0.7 5.1
cPARP/β-actin
55
40
β-actin

Fig 3C

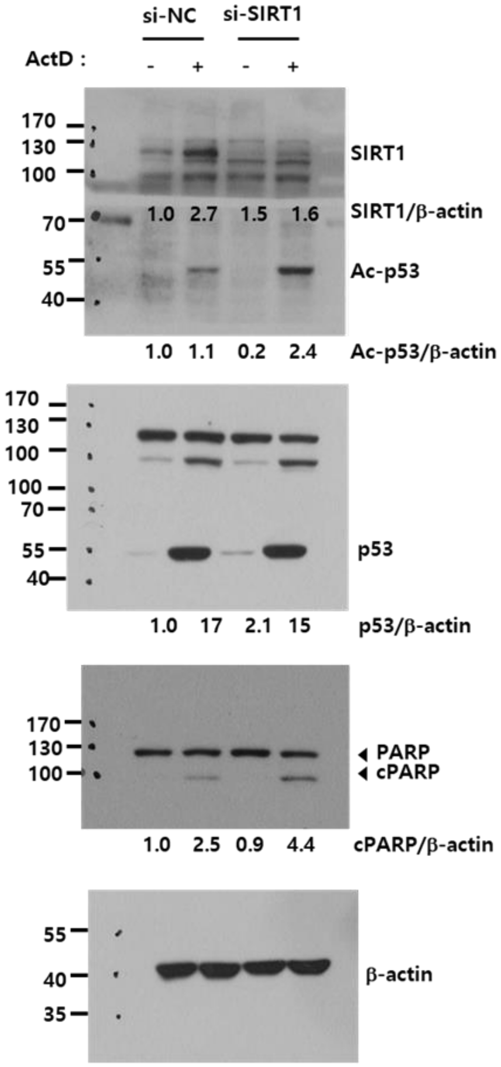
si-NC
si-SIRT1
ActD : - + - +
170
130
100
70
55
40
SIRT1
1.0 2.7 1.5 1.6
SIRT1/β-actin
Ac-p53
1.0 1.1 0.2 2.4
Ac-p53/β-actin
170
130
100
100
70
55
40
p53
1.0 17 2.1 15
p53/β-actin
170
130
100
PARP
cPARP
1.0 2.5 0.9 4.4
cPARP/β-actin
55
40
35
β-actin

Fig 3E

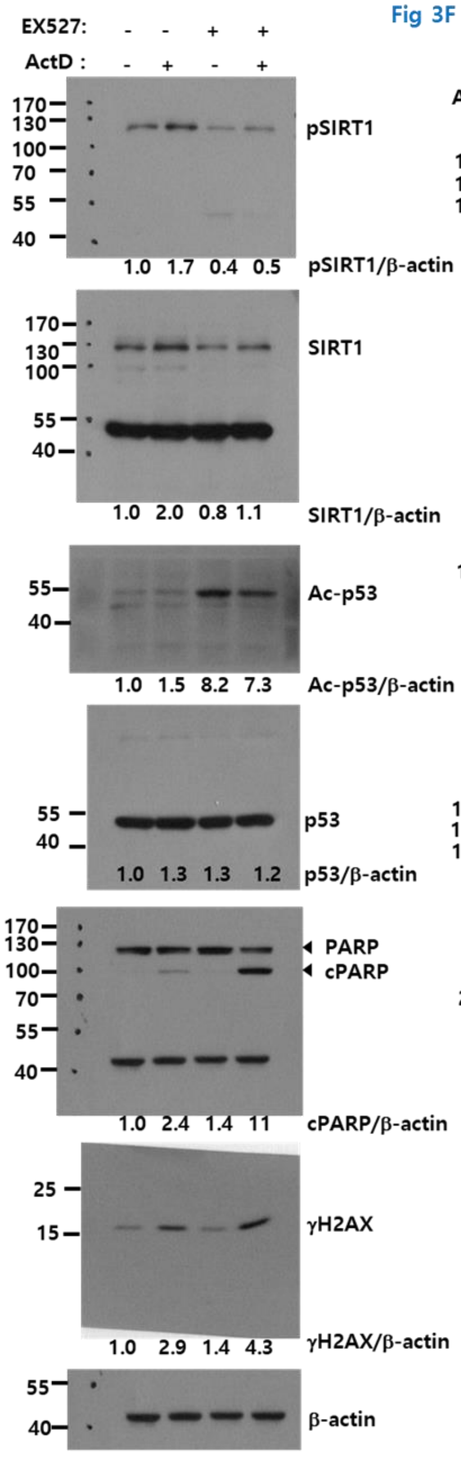
EX527: - - + +
ActD : - + - +
170
130
100
70
55
40
pSIRT1
1.0 1.7 0.4 0.5 pSIRT1/β-actin
SIRT1
1.0 2.0 0.8 1.1 SIRT1/β-actin
Ac-p53
1.0 1.5 8.2 7.3 Ac-p53/β-actin
p53
1.0 1.3 1.3 1.2 p53/β-actin
PARP
cPARP
1.0 2.4 1.4 11 cPARP/β-actin
25
15
γH2AX
1.0 2.9 1.4 4.3 γH2AX/β-actin
β-actin

Fig 3F

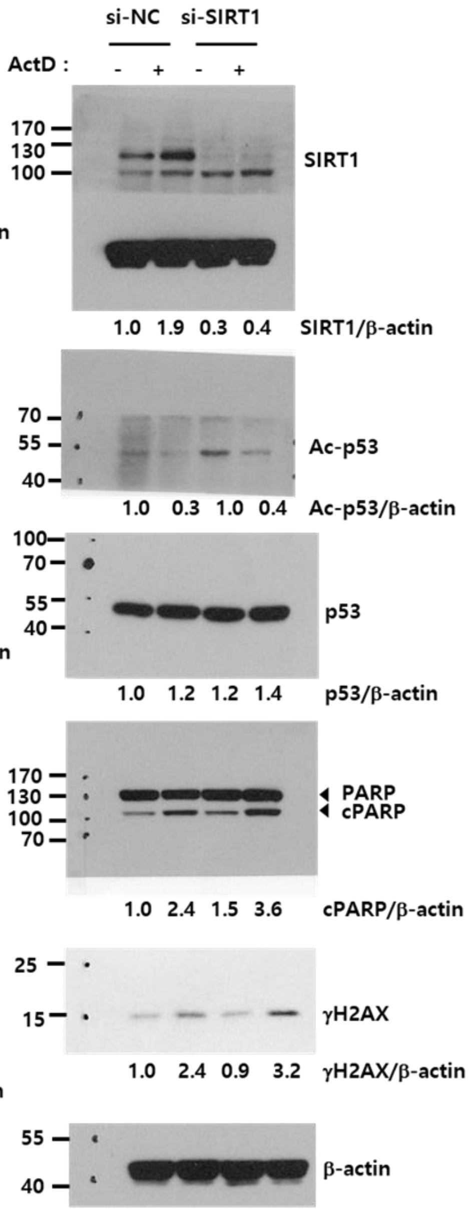
si-NC si-SIRT1
ActD : - + - +
SIRT1
1.0 1.9 0.3 0.4 SIRT1/β-actin
Ac-p53
1.0 0.3 1.0 0.4 Ac-p53/β-actin
p53
1.0 1.2 1.2 1.4 p53/β-actin
PARP
cPARP
1.0 2.4 1.5 3.6 cPARP/β-actin
γH2AX
1.0 2.4 0.9 3.2 γH2AX/β-actin
β-actin

Fig 4A

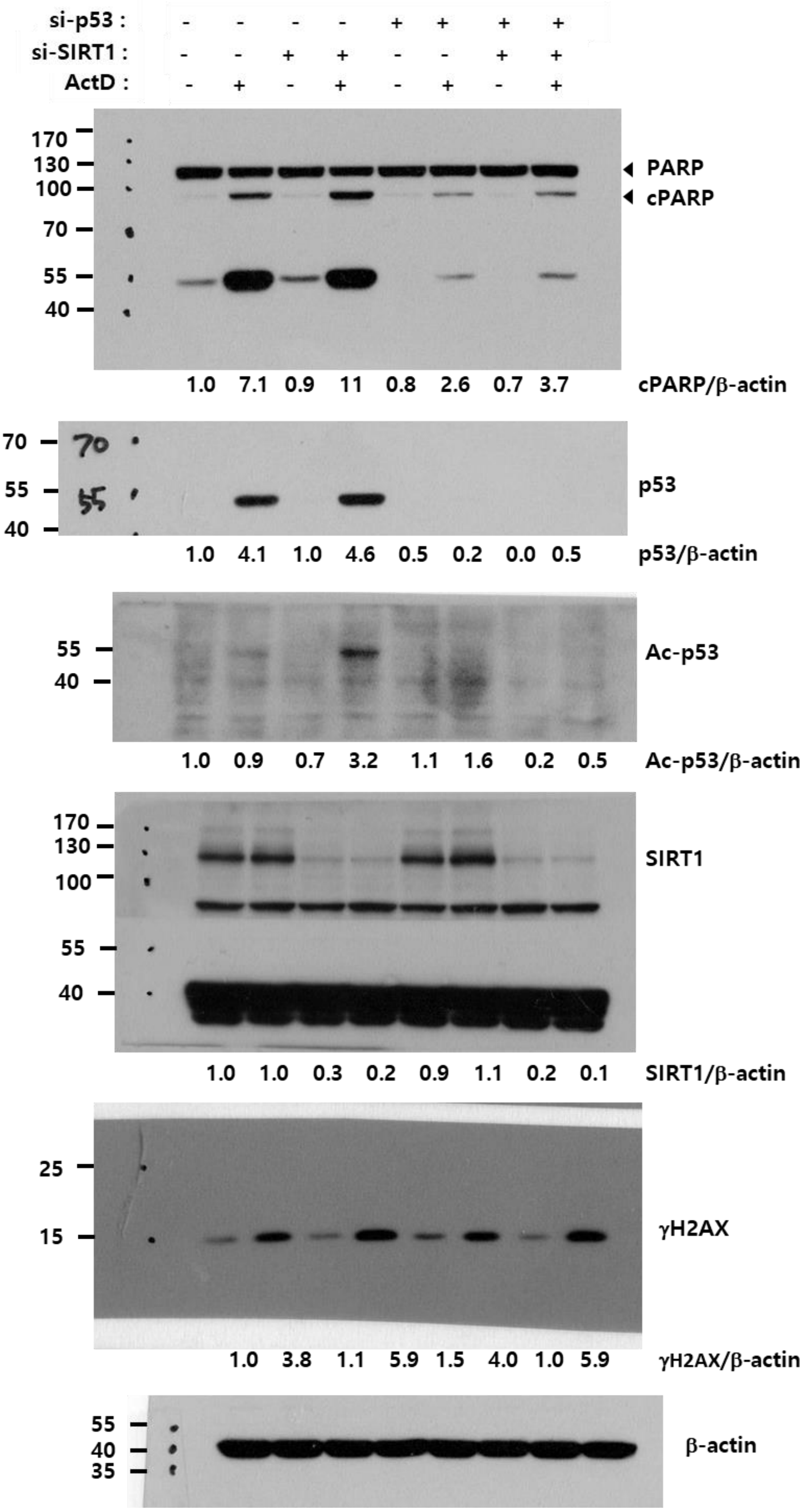
si-p53 : - - - - + + + +
si-SIRT1 : - - + + - - + +
ActD : - + - + - + - +
170
130
100
70
55
40
PARP
cPARP
1.0 7.1 0.9 11 0.8 2.6 0.7 3.7
cPARP/β-actin
70
55
40
p53
1.0 4.1 1.0 4.6 0.5 0.2 0.0 0.5
p53/β-actin
55
40
Ac-p53
1.0 0.9 0.7 3.2 1.1 1.6 0.2 0.5
Ac-p53/β-actin
170
130
100
55
40
SIRT1
1.0 1.0 0.3 0.2 0.9 1.1 0.2 0.1
SIRT1/β-actin
25
15
γH2AX
1.0 3.8 1.1 5.9 1.5 4.0 1.0 5.9
γH2AX/β-actin
55
40
35
β-actin

Fig 4C

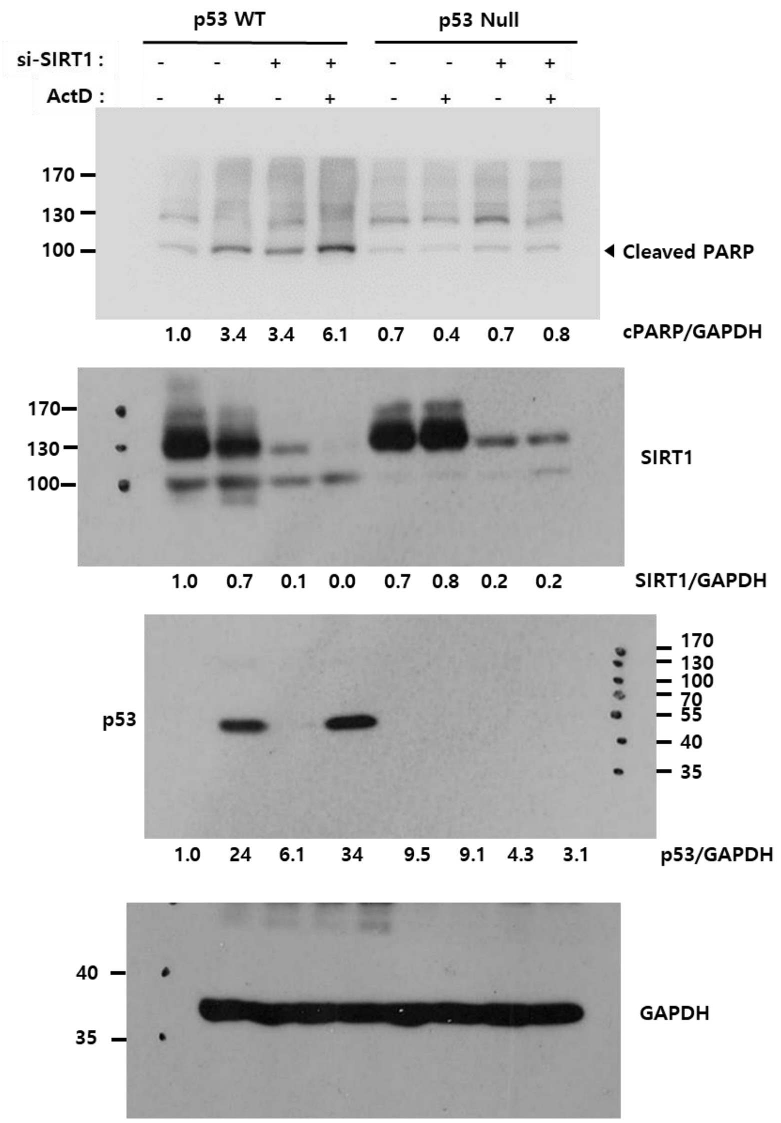
p53 WT
p53 Null
si-SIRT1 : - - + + - - + +
ActD : - + - + - + - +
170
130
100
Cleaved PARP
1.0 3.4 3.4 6.1 0.7 0.4 0.7 0.8
cPARP/GAPDH
170
130
100
SIRT1
1.0 0.7 0.1 0.0 0.7 0.8 0.2 0.2
SIRT1/GAPDH
p53
170
130
100
70
55
40
35
1.0 24 6.1 34 9.5 9.1 4.3 3.1
p53/GAPDH
40
35
GAPDH

Fig 5A

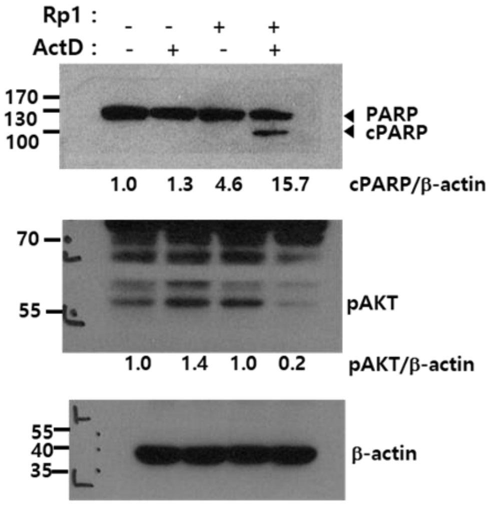

Rp1 : - - + +
ActD : - + - +
170
130
100
PARP
cPARP
1.0 1.3 4.6 15.7
cPARP/β-actin
70
55
pAKT
1.0 1.4 1.0 0.2
pAKT/β-actin
55
40
35
β-actin

Fig 5C

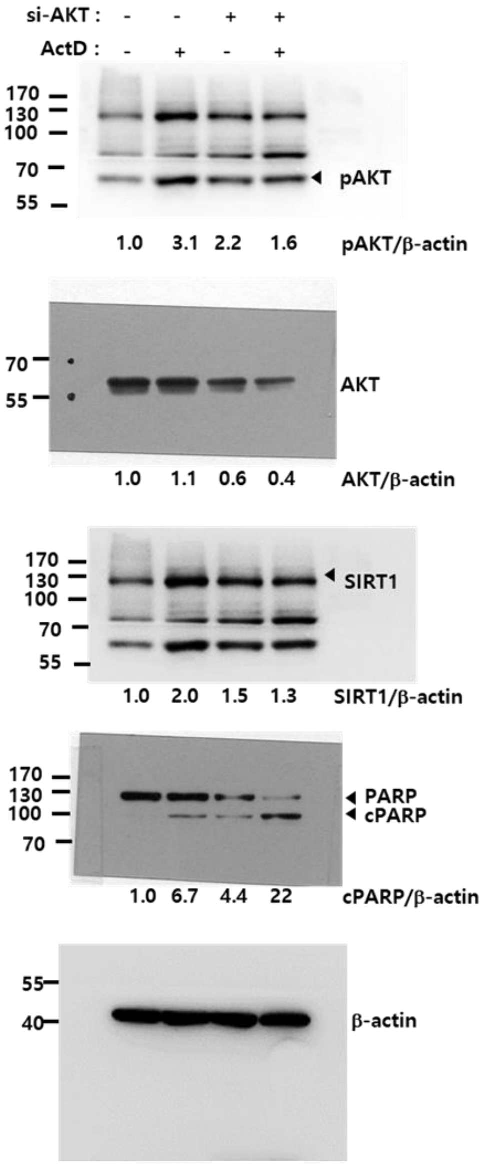

si-AKT : - - + +
ActD : - + - +
170
130
100
70
55
pAKT
1.0 3.1 2.2 1.6
pAKT/β-actin
70
55
AKT
1.0 1.1 0.6 0.4
AKT/β-actin
170
130
100
70
55
SIRT1
1.0 2.0 1.5 1.3
SIRT1/β-actin
170
130
100
70
PARP
cPARP
1.0 6.7 4.4 22
cPARP/β-actin
55
40
β-actin

Fig 5E

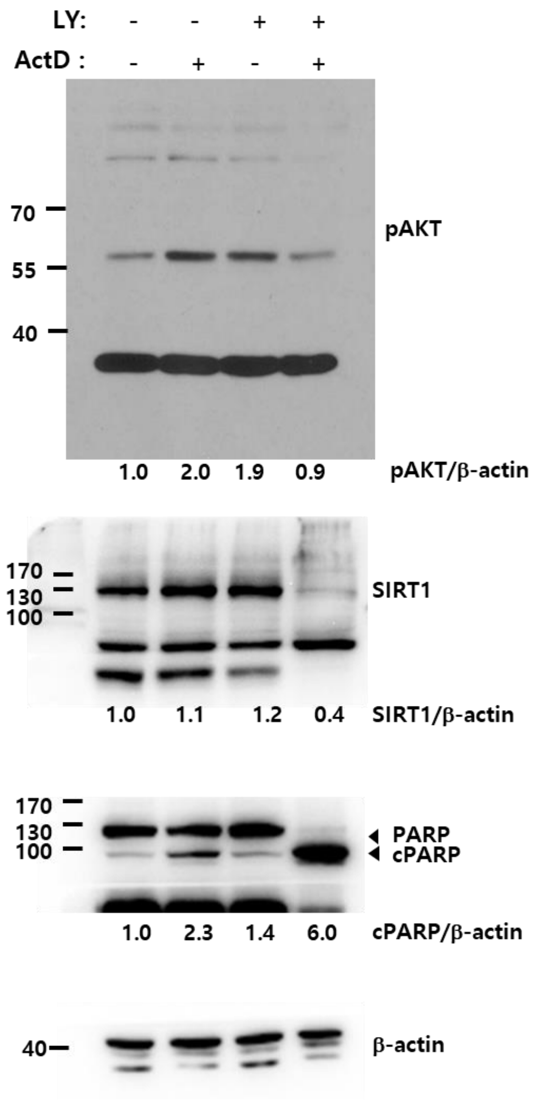
LY: - - + +
ActD : - + - +
70
55
40
pAKT
1.0 2.0 1.9 0.9
pAKT/β-actin
170
130
100
SIRT1
1.0 1.1 1.2 0.4
SIRT1/β-actin
170
130
100
PARP
cPARP
1.0 2.3 1.4 6.0
cPARP/β-actin
40
β-actin

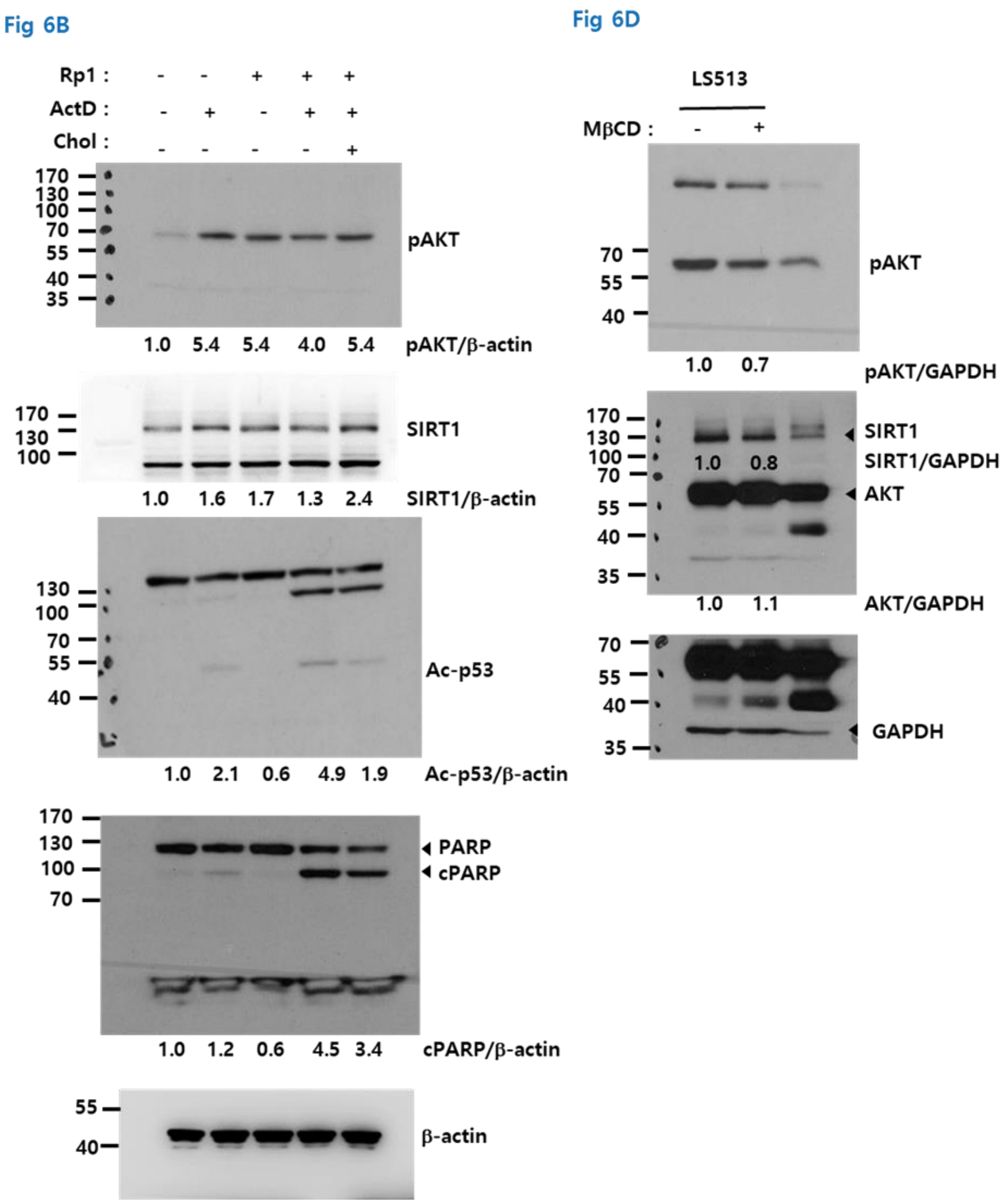

Figure S3. Original western blots.

© 2020 by the authors. Licensee MDPI, Basel, Switzerland. This article is an open access article distributed under the terms and conditions of the Creative Commons Attribution (CC BY) license (http://creativecommons.org/licenses/by/4.0/).
